# Dihydroartemisinin Inhibits Glucose Uptake and Cooperates with Glycolysis Inhibitor to Induce Apoptosis in Non-Small Cell Lung Carcinoma Cells

**DOI:** 10.1371/journal.pone.0120426

**Published:** 2015-03-23

**Authors:** Yan-jun Mi, Guo-jun Geng, Zheng-zhi Zou, Jing Gao, Xian-yang Luo, Yu Liu, Ning Li, Chun-lei Li, Yu-qiang Chen, Xiu-yi Yu, Jie Jiang

**Affiliations:** 1 Department of thoracic surgery, The First Affiliated Hospital of Xiamen University, Xiamen, China; 2 Department of Medical Oncology, Chenggong Hospital of Xiamen University, Xiamen, China; 3 MOE Key Laboratory of Laser Life Science and Institute of Laser Life Science, College of Biophotonics, South China Normal University, Guangzhou, China; 4 Department of Head and Neck Surgery, The First Affiliated Hospital of Xiamen University, Xiamen, China; Indiana University, UNITED STATES

## Abstract

Despite recent advances in the therapy of non-small cell lung cancer (NSCLC), the chemotherapy efficacy against NSCLC is still unsatisfactory. Previous studies show the herbal antimalarial drug dihydroartemisinin (DHA) displays cytotoxic to multiple human tumors. Here, we showed that DHA decreased cell viability and colony formation, induced apoptosis in A549 and PC-9 cells. Additionally, we first revealed DHA inhibited glucose uptake in NSCLC cells. Moreover, glycolytic metabolism was attenuated by DHA, including inhibition of ATP and lactate production. Consequently, we demonstrated that the phosphorylated forms of both S6 ribosomal protein and mechanistic target of rapamycin (mTOR), and GLUT1 levels were abrogated by DHA treatment in NSCLC cells. Furthermore, the upregulation of mTOR activation by high expressed Rheb increased the level of glycolytic metabolism and cell viability inhibited by DHA. These results suggested that DHA-suppressed glycolytic metabolism might be associated with mTOR activation and GLUT1 expression. Besides, we showed GLUT1 overexpression significantly attenuated DHA-triggered NSCLC cells apoptosis. Notably, DHA synergized with 2-Deoxy-D-glucose (2DG, a glycolysis inhibitor) to reduce cell viability and increase cell apoptosis in A549 and PC-9 cells. However, the combination of the two compounds displayed minimal toxicity to WI-38 cells, a normal lung fibroblast cell line. More importantly, 2DG synergistically potentiated DHA-induced activation of caspase-9, -8 and -3, as well as the levels of both cytochrome c and AIF of cytoplasm. However, 2DG failed to increase the reactive oxygen species (ROS) levels elicited by DHA. Overall, the data shown above indicated DHA plus 2DG induced apoptosis was involved in both extrinsic and intrinsic apoptosis pathways in NSCLC cells.

## Introduction

Lung cancer is the most common malignant tumor and the leading cause of cancer-related mortality worldwide. Non-small cell lung cancer (NSCLC) is the most common type of lung cancer. Resistance of NSCLC cells to apoptosis is a major obstacle in anticancer treatment. Accordingly, current researches focus on the development of innovative compounds that promote the apoptosis of therapy-resistant NSCLC cells. Dihydroartemisinin (DHA) is an important derivative of Artemisinin, a natural product isolated from Chinese medicinal herb *Artemisia annua* L. (qinghao). As a very potent anti-malarial drug, DHA has been used as first-line therapeutics against malaria falciparum worldwide. Recently, studies have shown that DHA has profound effect against breast cancer [1], papillomavirus-expressing cervical cancer [2], liver cancer and pancreatic cancer [3,4]. Additionally, DHA has been shown to exert anticancer effects by induction of apoptosis without obvious side effects in lung carcinomas [5]. Moreover, ionizing radiation potentiates DHA-induced NSCLC cells apoptosis [6]. Apart from its prominent pro-apoptotic effect, DHA affects cancer cell functions, including tumor cell proliferation [7], angiogenesis [8], and immune regulation [9]. However, the exact molecular mechanisms of DHA anticancer effects remain to be fully investigated.

A unique characteristic of many tumor cells is increased glucose uptake and elevated aerobic glycolysis. Glycolysis with generation of lactate and reduced mitochondrial oxidative phosphorylation metabolism through the tricarboxylic acid (TCA) cycle is commonly found in cancer cells. This remarkable metabolic reprogramming, known as the Warburg effect [10,11], provides cancer cells an advantage to grow even in regions with hypoxia. Therefore, the especial dependence of cancer cells on glycolysis makes them vulnerable to therapeutic intervention with specific glycolysis target inhibitors [12,13]. The glycolytic inhibitor 2-Deoxy-D-glucose (2DG), targeting hexokinase which is the entry-point enzyme for glycolysis [14], has been studied as a promising therapeutic compound that targets metabolic alterations of tumor cells [15,16]. Some pieces of evidences suggest that targeting glycolysis could be a good strategy against NSCLC [12]. These NSCLC cells treated with glycolysis inhibitor 2DG display mitochondrial respiratory defects and increased apoptosis [17].

In the current study, we showed that DHA inhibited cell proliferation and colony formation, induced cell apoptosis in cultured human NSCLC cells. Furthermore, we provided evidences that DHA inhibited glucose uptake and ATP production and decreased lactate content in NSCLC cells. In addition, we found that DHA inhibited glucose uptake linked to inhibition of mTOR activity and reduction of glucose transporter 1 (GLUT1) expression. Moreover, we showed the combination of DHA and 2DG was synergistic at inhibiting cell proliferation and inducing apoptosis in NSCLC cells. Lastly, we indicated that DHA combined with 2DG induced cell apoptosis was involved in mitochondrial-mediated pathway and caspase-8-dependent pathway.

## Materials and Methods

### Cell culture, reagents and drug treatment

A549, PC-9 and WI-38 cell lines were obtained from the American Type Culture Collection (ATCC) and grown in DMEM medium (Gibco, Life Technologies, Carlsbad, CA) supplemented with 10% (v/v) fetal bovine serum (FBS) (Gibco, Life Technologies, Carlsbad, CA) and 4.5g/L glucose (24.75 mM) at 37°C in 5% CO_2_ incubator. Cells were grown in monolayer and passaged routinely 2–3 times a week. DHA was purchased from Selleck Chemicals LLC (Houston TX, USA). MTT (3-(4,5-Dimethylthiazol-2-yl)-2,5-diphen​yltetrazolium bromide), and dimethyl sulfoxide (DMSO), glucose, 2-Deoxy-D-glucose (2DG) and DMEM free glucose medium were purchased from Sigma (St. Louis, MO, USA). For drug treatment, DHA and 2DG were dissolved in DMSO and sterilized distilled water respectively; aliquots were stored at -80°C. Stock solutions were diluted to the desired final concentrations with growth medium just before use. Cells were seeded in triplicate at a density of 0.1–0.2 million/well in six well plates. Prior to drug treatment, cells were incubated for at least 12 h and thereafter replaced with media containing drugs, followed by 24 or 48 h incubation. DMSO-treated cells were used as a mock control.

### Cell viability, clonogenic cell survival and apoptosis assays

Cell viability was assessed by standard MTT assay. The optical density at 490 nm was measured using a multi-well plate reader (Micro-plate Reader, Bio-Rad, Hercules, CA). For clonogenic cell survival experiments, the attached cells from the same dish that were trypsinized with 1 mL trypsin–EDTA (Life Technologies, Carlsbad, CA) and inactivated with media containing 10% FBS. The cells were diluted and counted using a hemocytometer. Cells were plated at low density (200 per well in six well plate). After 24 h, drugs were added at indicated concentration for 48 h. After drugs removal, cells were allowed to proliferate, and clones were allowed to grow in a humidified 5% CO_2_, 37°C environment for 15 days in growth medium. Cells were fixed with 70% ethanol and stained with 0.05% crystal violet (sigma, St. Louis, MO, USA) for analysis of clonogenic cell survival as previously described [18]. Measurement of apoptosis was conducted by Annexin V-FITC (fluorescein isothiocyanate)/propidium iodide (PI) analysis as described previously [19]. Apoptosis analysis was conducted using the Apoptosis Detection Kit (Beyotime Institute of Biotechnology, Jiangsu, China) following the manufacturer’s protocol. Briefly, cells were seeded and treated with the drugs for 24 and 48 h. Afterward, the cells were washed twice with PBS and 1×10^6^ cells were resuspended in 1 mL of 1×Annexin V binding buffer. Cells undergoing apoptotic cell death were analyzed by counting the cells that stained positive for Annexin V/FITC and negative for PI, and late stage of apoptosis as Annexin V/FITC and PI positive using FACS Calibur (BD Biosciences, New Jersey, USA).

### Measurements of glucose levels and lactate production of cells in the media

Per well of a 12-well plate was seeded with a total of 3×10^5^ cells treated with various drugs. Cells were counted before measurements using trypan blue staining. For assessment of glucose uptake, the media were collected and the glucose was immediately measured using an Olympus AU5400 (Olympus Corporation, Tokyo, Japan). For assessment of lactate production, the media was collected and diluted 1:100 in lactate assay buffer. The amount of lactate present in the media was then estimated using the Lactate Assay Kit (sigma, St. Louis, MO, USA) according to the manufacturer’s instructions.

### ATP assay

Cell ATP content was determined over time using the ATP Bioluminescent Somatic Cell Assay kit (sigma, St. Louis, MO, USA) according to the manufacturer’s recommendations. Briefly, cells were seeded and treated with the drugs for 24 h. Subsequently, the cells were washed twice with PBS and were lysed on ice with somatic cell ATP releasing reagent. Then the cell lysis to be assayed was mixed with solution including luciferase. Swirl briskly, transfer 0.1 mL to the reaction vial, and immediately measure the amount of light emitted with a luminometer (Charm Sciences, Malden, MA).

### Western blot analysis

Cells were lysed on ice in RIPA buffer. The protein concentration was determined by Bradford dye method. Equal amounts (20 to 40 μg) of cell extract were subjected to electrophoresis in 6–12.5% sodium dodecyl sulfate-polyacrylamide (SDS-PAGE) and transferred to PVDF membranes (Millipore, Darmstadt, Germany) for antibody blotting. The membranes were blocked and then incubated with p-mTOR (Ser2448), mTOR, p-S6 (S235/236), S6, AIF and actin antibodies (all from Cell Signaling Technologies, Massachusetts, USA). And the GLUT1 and GLUT4 antibodies were purchased form Abcam (Cambridge, U.K). Monoclonal antibodies against cytochrome c and oxidase subunit IV (COX IV) were purchased from BD PharMingen. Subsequently, the membranes were incubated with a HRP-conjugated secondary antibody (Protein Tech Group, Chicago, IL) at room temperature for 1 h. Then the membranes visualized using Dura SuperSignal Substrate (Pierce, USA), according to the manufacturer’s instructions. Mitochondrial isolation was performed with a mitochondrial isolation kit (KeyGEN).

### Glucose starvation assay

A549 and PC-9 cell lines were cultured in DMEM free glucose medium supplemented with 10% FBS, 0–10 mM glucose and DHA (20 μM for A549 cells and 16 μM for PC-9 cells) for 48 h.

### Combination Index

For combination treatment of DHA and/or 2DG, MTT assay data were converted to fraction of growth affected by the individual drug or the combination treated NSCLC cells compared with untreated cells and analysed using CalcuSyn software (Biosoft, Ferguson, MO, USA) to determine whether the combination was synergistic. This program is based upon the Chou–Talalay equation, which calculates a combination index (CI) [20]. The general equation for the classic isobologram is given by: CI = (D)_1_/(Dx)_1_ + (D)_2_/(Dx)_2_. Where Dx indicates the dose of one compound alone required to produce an effect, (D)_1_ and (D)_2_ are the doses of compounds 1 and 2, respectively, necessary to produce the same effect in combination. From this analysis, the combined effects of the two agents can be summarized as follows: CI < 1, CI = 1, CI > 1 indicate synergistic, additive and antagonistic effects, respectively, as described previously [18].

### Confocal microscopy imaging of living cells

A549 and PC-9 cells were treated with indicated concentrations of DHA and 2DG for 48 h. Imaging of Living Cells was performed on a confocal microscope (LSM510/ConfoCor2, Zeiss, Jena, Germany) with a 40×oil immersion plan apochromat objective lens. Images were recorded using a digital camera with 1280×1280 pixels resolution.

### Plasmid transfection

Cells were transfected with GLUT1 constructs (Addgene, 18085) [21], and Rheb construts (Genecopoeia, EX-A0963-Lv105–5). In brief, A549 and PC-9 cells were seeded at a density of 8×10^5^ cells per well in 6 well plate. After 8 h, cells were placed in 1 mL of plasmid mixture with 2 μg plasmid and 5 μL lipofectamine 2000 (Life Technologies, Carlsbad, CA) according to the manufacturer's protocol. After 8 h of transfection, 1 mL of RPMI-1640 complete medium was added, and experiments were conducted 48 h after transfection.

### Caspase activity assay

Fluorometric assays of caspase activity were carried out by using the substrate Ac-DEVD-AMC (BD Pharmingen, San Diego, CA) for caspase-3, Ac-LHED-AMC (BD Pharmingen, San Diego, CA) for caspase-9 and Ac-IETD-AMC (BD Pharmingen, San Diego, CA) for caspase-8. Briefly, cells were lysed in lysis buffer (10 mM HEPES, 142 mM KCl, 5 mM MgCl_2_, 1 mM EDTA, 0.2% NP-40 and pH 7.2) with 10 mM DTT. Following incubation for 30 min on ice, samples were centrifuged at 12,000 rpm for 30 min at 4°C and the protein content in supernatants was determined by Bradford dye method. Aliquots of 10 mg/100 mL assay volume were incubated with 140 mM site-specific tetrapeptide substrates Ac-DEVD-AMC for caspase-3 and Ac-IETD-AMC for caspase-8 in a caspase assay buffer (20 mM HEPES, 100 mM NaCl, 1 mM EDTA, 0.01% (w/v) CHAPS, 10% (w/v) sucrose and pH 7.2) with 10 mM DTT for 30 min. The release of the fluorogenic group AMC was determined at 37°C in a VersaFluor Fluorometer (Bio-Rad, Hercules, CA) with excitation at 380 nm and emission at 440 nm.

### Measurement of intracellular reactive oxygen species (ROS) generation

ROS generation inside living cells was measured by FCM with 29,79-Dichlorodihydrofluorescein diacetate (DCFH-DA) (Wako Ltd, Osaka, Japan), an oxidation-sensitive probe, which is cleaved by nonspecific esterases and turns to highly fluorescent DCF upon oxidation by ROS. Untreated or treated cells were stained with 20 μM DCFH-DA for 30 min at 37°C in the dark and subsequently assayed by FCM as described in detail previously [12].

### Statistics

All experiments were repeated three times and were expressed as mean ± SD. *P* values were calculated using student’s *t* test and *P* value < 0.05 was considered significant. Statistical analysis was analyzed using the Statistical Package for Social Sciences (SPSS) software (version 16.0).

## Results

### DHA inhibits cell viability and colony formation and induces apoptosis in NSCLC cells

**Fig 1:**
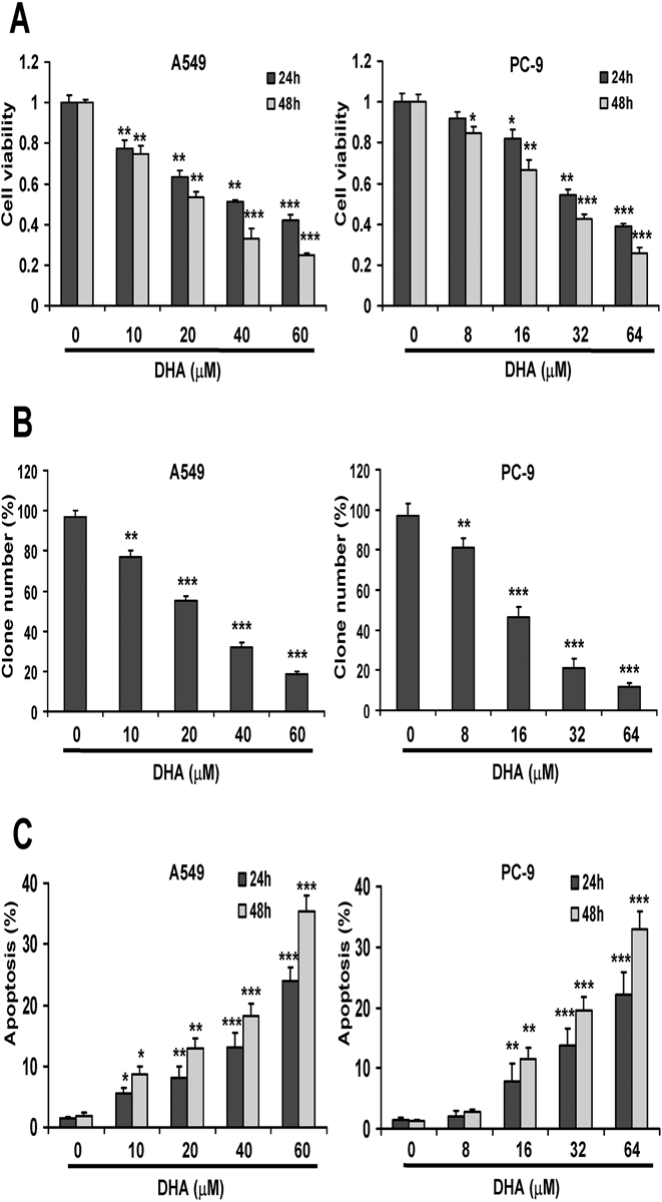
DHA inhibits cell viability and colony formation and induces apoptosis in NSCLC cells. (A) DHA-induced concentration-dependent reduction of cell viability in A549 and PC-9 cells. Cells were incubated with indicated concentrations of DHA for 24 h and 48 h. Cell growth inhibition activity of DHA was assessed by MTT. (B) Influence of A549 and PC-9 cells on the number of colony-forming cells, as evaluated by clonogenic assay. Cells were incubated with indicated concentrations of DHA for 48 h, and then media were replaced with fresh growth media and cultured for additional 15 days. The clonogenic assay was done as described in Materials and Methods. (C) Influence of DHA on apoptosis in A549 and PC-9 cells. Cells were treated with indicated concentrations of DHA for 24 h and 48 h. Cell apoptosis was assessed by Annexin V-FITC/PI staining assay. Columns, mean of three determinations; bars, SD. *, *P* < 0.05; **, *P* < 0.01; ***, *P* < 0.001, control versus DHA-treated cells.

To determine the effects of DHA on human NSCLC cell viability, A549 and PC-9 cells were exposed to different concentrations of DHA for up to 24 h and 48 h, followed by the determination of cell viability using MTT assay. As shown in Fig. 1A, DHA caused a concentration- and time-dependent inhibition of cell viability with IC_50_ values of 42.2 μM (24 h) and 25.1 μM (48 h) in A549 cells, and IC_50_ values of 35.9 μM (24 h) and 25.3 μM (48 h) in PC-9 cells. Furthermore, A549 and PC-9 cells exposed to DHA were evaluated for their clonogenic potential. As shown in Fig. 1B, DHA reduced the clonogenic survival of A549 and PC-9 cells in a dose-dependent manner. To determine if the cytotoxic effect of DHA was due to induction of apoptosis, A549 and PC-9 cells were treated with different concentration of DHA for 24 h and 48 h and then cell apoptosis was determined by Annexin V-FITC and propidium iodide (PI) staining and flow cytometry analysis. As shown in Fig. 1C, DHA induced cell apoptosis in the two cell lines in a dose-dependent manner.

### DHA decreases the level of glycolytic metabolism in NSCLC cells

**Fig 2:**
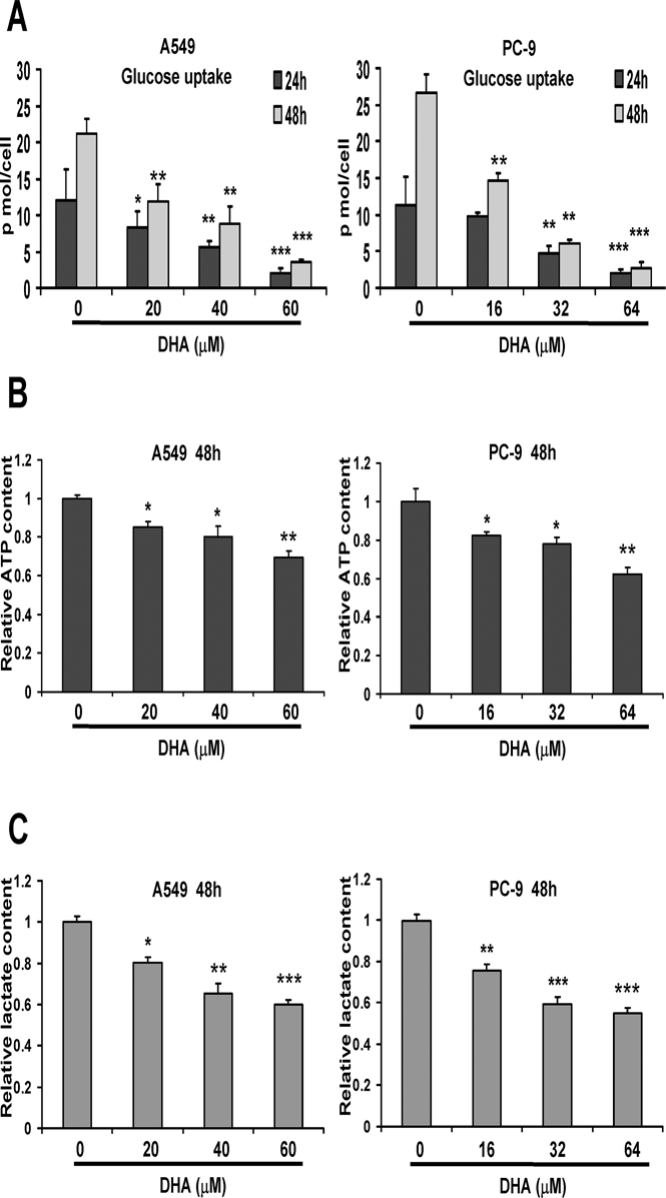
DHA decreases the level of glycolytic metabolism in NSCLC cells. (A) A549 and PC-9 cells were treated with indicated concentrations of DHA for 24 h and 48 h. After 24 h and 48 h, the cells were counted and the glucose in the culture media was immediately tested. Then the results were normalized to the number of cells, and performed as pmol/cell. (B) A549 and PC-9 cells were treated with indicated concentrations of DHA for 48 h. After 48 h, relative ATP content was determined using a bioluminescence assay. Then the results were normalized to the number of cells. (C) A549 and PC-9 cells were treated with indicated concentrations of DHA for 48 h. After 48 h, relative lactate content was determined as described in Materials and Methods. Then the results were normalized to the number of cells. Columns, mean of three determinations; bars, SD. *, *P* < 0.05; **, *P* < 0.01; ***, *P* < 0.001, control versus DHA-treated cells.

**Fig 3:**
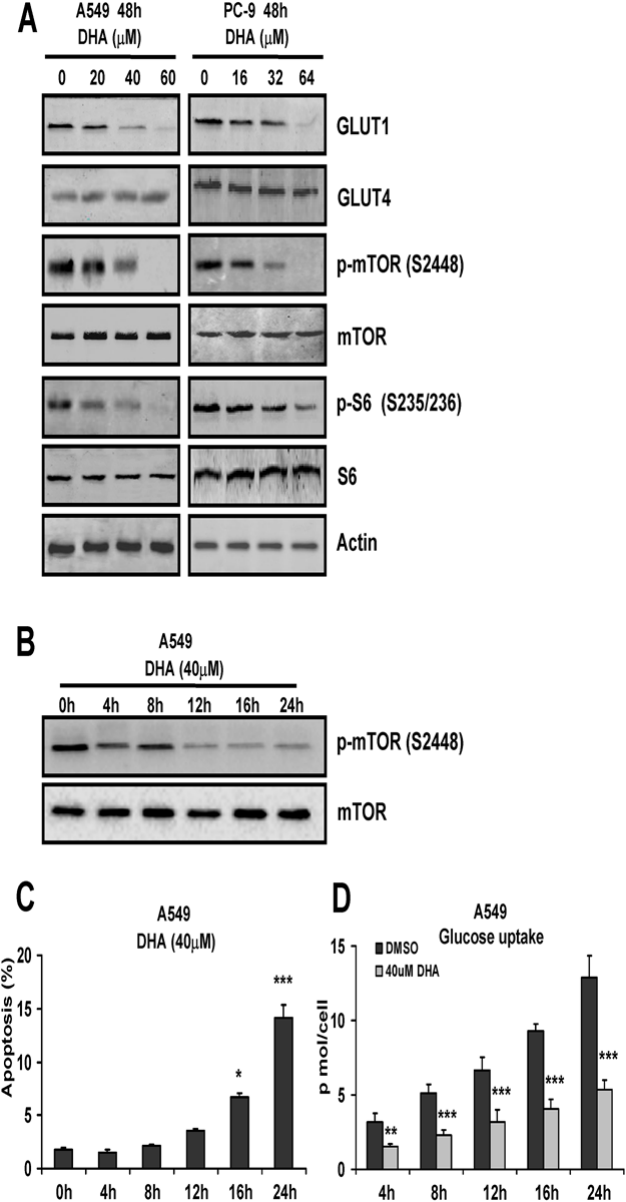
DHA-suppressed glycolytic metabolism is associated with mTOR activation and GLUT1 expression. (A) A549 and PC-9 cells were treated with indicated concentrations of DHA for 48 h, (B) A549 cells were treated with 40 μM DHA for indicated time, respectively, and then cell lysates were subjected to Western blot analysis. (C) A549 cells were treated with 40 μM DHA for indicated time, respectively, cell apoptosis was assessed by Annexin V-FITC/PI staining assay. (D) A549 cells were treated with 40 μM DHA for indicated time, respectively, and then the cells were counted and the glucose in the culture media was immediately tested. Then the results were normalized to the number of cells, and performed as p mol/cell. Columns, mean of three determinations; bars, SD. *, *P* < 0.05; **, *P* < 0.01; ***, *P* < 0.001, control versus DHA-treated cells.

We next assessed whether DHA showed any effect on glucose transport in NSCLC cells. A549 and PC-9 cells were treated with different concentrations of DHA, respectively. Twenty-four and forty-eight hours later, glucose uptake was detected. As shown in Fig. 2A and 3B, DHA inhibited the glucose uptake in A549 and PC-9 cells in a time- and dose-dependent manner. To determine whether DHA resulted in reduced glycolysis, we measured ATP and lactate levels after DHA treatment. The two cell lines were treated with different concentrations of DHA respectively for 48 h, and then cell ATP content and lactate levels were detected. We found that both ATP and lactate levels were decreased significantly after DHA treatment in NSCLC cells in a dose-dependent manner (Fig. 2B and 2C). All these results indicated that DHA suppressed glycolysis in NSCLC cells.

### DHA-suppressed glycolytic metabolism is associated with inhibition of mTOR activation and GLUT1 expression

Glycolytic metabolism is regulated by a number of kinases including mTOR, which is generally thought to be involved in the positive control of glycolytic metabolism. To provide insights into the mechanisms of action and targets of DHA, protein expression levels of *mTOR* pathway molecules were examined by Western blotting in NSCLC cells after treatment with this drug. As shown in Fig. 3A, after a 48 h treatment with DHA in A549 and PC-9 cells, we found significant decreases in the levels of phosphorylated mTOR and its downstream signalling molecule phospho-S6 ribosomal protein. Furthermore, the levels of phosphorylated mTOR were analysed at various time points (0–24 h) in A549 cells treated with 40 μM DHA. As shown in Fig. 3B, the phosphorylated mTOR was decreased in a time-dependent manner. In addition, we determined the effects of DHA on GLUT1 and GLUT4 expression. As shown in Fig. 3A, we found DHA decreased remarkably the GLUT1 expression. However, there were no significant changes in GLUT4 expression in NSCLC cells treated with DHA (Fig. 3A). Further the cell apoptosis and glucose uptake levels were determined in A549 cells treated with 40 μM DHA for 0–24 h. The results showed that only 4 h exposure to DHA was sufficient to inhibit glucose uptake in A549 cells, however longer continuous exposure times (>24 h) were required for commitment to cell apoptosis in the cells exposed to equivalent dose of DHA (Fig. 3C and D). This implied the attenuation of glycolytic metabolism was not caused by cell apoptosis. All these results hinted that mTOR inhibition by DHA resulted in decreased glucose uptake and glycolytic metabolism through inhibition of GLUT1 expression.

### Upregulated mTOR activation increases the level of glycolytic metabolism and cell viability inhibited by DHA

**Fig 4:**
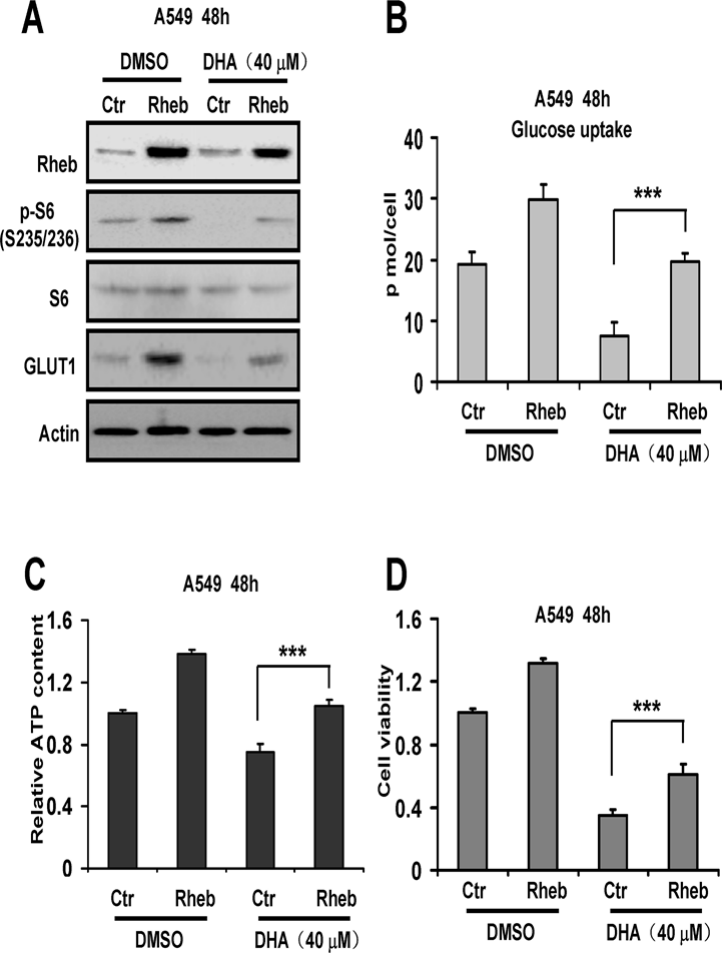
Upregulated mTOR activation increased the level of glycolytic metabolism and cell viability inhibited by DHA. A549 cells were transfected with control (Ctr) and Rheb vector for 8 h respectively, and then treated with the indicated concentration of DHA for 48 h. (A) Cell lysates were subjected to Western blot analysis with the indicated antibodies. (B) The cells were counted and the glucose in the culture media was immediately tested. Then the results were normalized to the number of cells, and performed as p mol/cell. (C) The relative ATP content was determined using a bioluminescence assay. Then the results were normalized to the number of cells. (D) Cell growth inhibition activity was assessed by MTT. The experiments were repeated thrice. Columns, mean; bars, SD. ***, *P* < 0.001.

The data shown above hinted that the reduction of glycolytic metabolism and cell viability in NSCLC cells might be associated with inhibition of mTOR activation. Thus we then asked if stimulated mTOR activation could rescue DHA-decreased glycolytic metabolism and cell viability in NSCLC cells. As shown in Fig. 4A, overexpression of Rheb increased the levels of phosphorylated S6 ribosomal protein and GLUT1 in A549 cells. These results suggested Rheb activated mTOR signaling. In addition, we also showed that the reduced expression of the two molecules induced by DHA was suppressed by Rheb (Fig. 4A). Moreover, overexpression of Rheb suppressed the reduction of glucose uptake and ATP content in DHA-treated A549 cells (Fig. 4B and C). In addition, Fig. 4D indicated that overexpression of Rheb prevented loss of cell viability induced by DHA in A549 cells. Thus, these results suggested that DHA increased cell viability, in part, by promoting glycolytic metabolism in A549 cells. Besides, these data further suggested that DHA-inhibited glycolytic metabolism was linked to mTOR signaling pathway in NSCLC cells.

### Overexpression of GLUT1 inhibits cell death triggered by DHA

**Fig 5:**
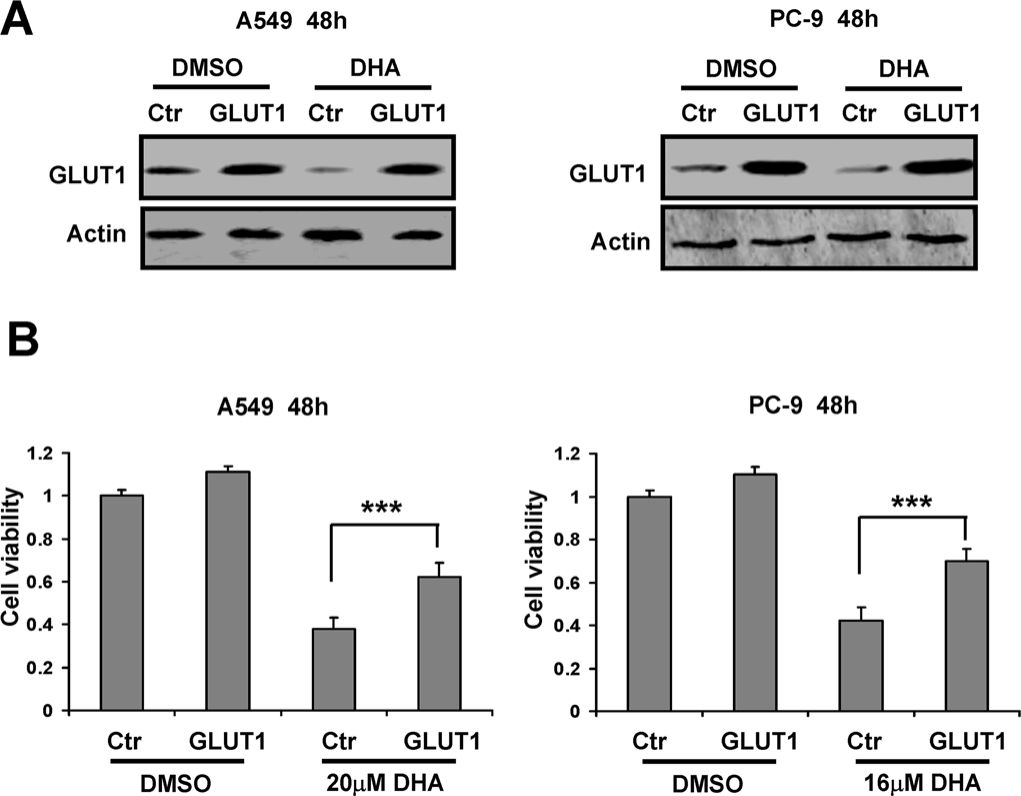
Overexpression of GLUT1 inhibits cell death triggered by DHA. A549 and PC-9 cells were transfected with control (Ctr) and GLUT1 vector for 8 h respectively, and then treated with the indicated concentration of DHA for 48 h. (A) Cell lysates were subjected to western blot analysis with the indicated antibodies. (B) Cell growth inhibition activity was assessed by MTT. This experiment was repeated thrice. Columns, mean; bars, SD. ***, *P* < 0.001.

The data shown above indicated that DHA-induced cytotoxicity in NSCLC cells was associated with inhibition of glucose uptake through down-regulation of GLUT1. Based on above findings, we then asked if overexpression of GLUT1 could rescue DHA-induced apoptosis in A549 and PC-9 cells. We overexpressed GLUT1 in A549 and PC-9 cells (Fig. 5A). Subsequently, the two cell lines were treated with 20 μM and 16 μM DHA for 48 h respectively. MTT assay showed that the survival rates of GLUT1 transfected cells were (62.11 ± 6.93% for A549 cells and 69.92 ± 5.73% for PC-9 cells), obviously higher than that of control empty vector transfected cells (38.5 ± 5.2% for A549 cells and 42.3 ± 6.56% for PC-9 cells) when treated with DHA (Fig. 5B). These results suggested overexpression of GLUT1 inhibited cell death triggered by DHA.

### Glucose prevents DHA-induced cytotoxicity in NSCLC cells

**Fig 6:**
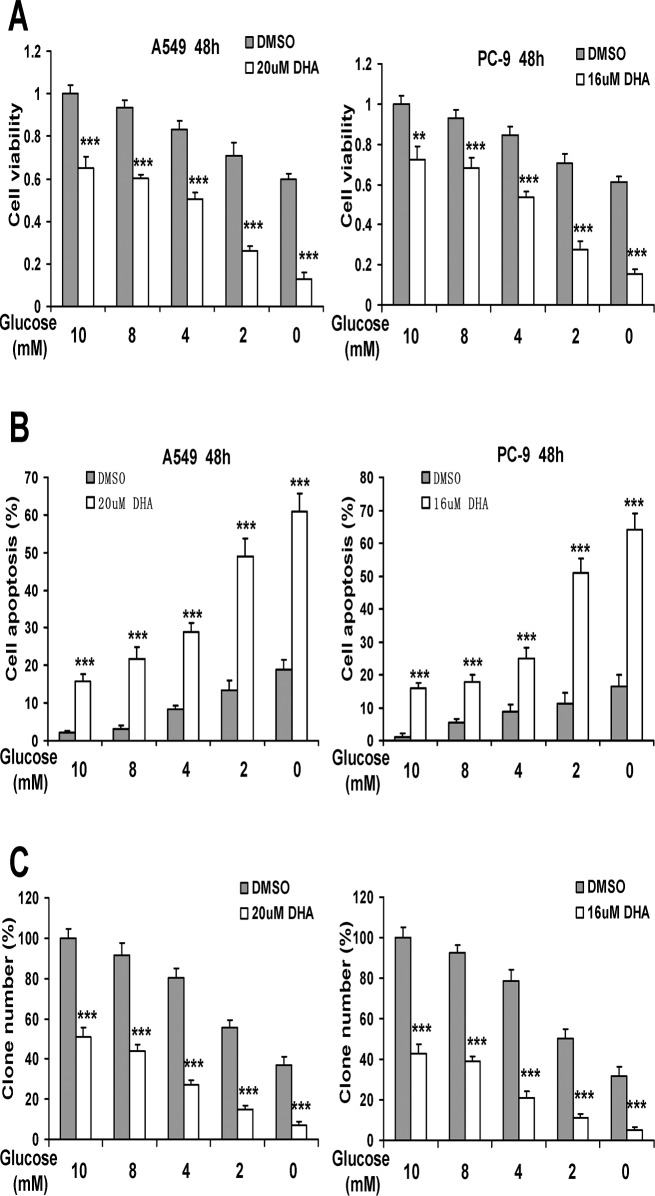
Glucose prevents DHA-induced cytotoxicity in NSCLC cells. (A and B) A549 and PC-9 cells were cultured with DMEM free glucose medium with different concentrations of glucose, and then treated with indicated doses of DHA for 48 h. Cell viability was measured using MTT assay and cell apoptosis was measured using an Annexin V-FITC/PI staining assay. (C) Cells were incubated with DMEM free glucose media containing indicated concentrations of DHA and glucose for 48 h, and then media were replaced with fresh growth media and cultured for additional 15 days. The clonogenic assay was done as described in Materials and Methods. Columns, mean of three determinations; bars, SD. *, *P* < 0.05; **, *P* < 0.01; ***, *P* < 0.001, DMSO versus DHA-treated cells.

The data shown above indicated that DHA-induced cytotoxicity might be involved in inhibition of glucose uptake and glycolytic metabolism in NSCLC cells. To test this hypothesis, we cultured A549 and PC-9 cells in different concentration of glucose medium (0–10 mM) to determine whether glucose deprivation could exaggerate the cytotoxicity effects of DHA. As shown in Fig. 6A, treatment with 20 μM DHA plus 10 mM glucose had weak effects on cell viability in A549 cells. However, there was a significantly lower cell viability found upon treatment with 20 μM DHA in the presence of decreasing concentrations of glucose. Similar results were found in PC-9 cells (Fig. 6A).

The data shown above indicated that glucose could prevent the cytotoxicity by DHA in A549 and PC-9 cells. We then asked if glucose could decrease DHA-induced apoptosis in NSCLC cells. A549 and PC-9 cells were cultured in different concentration of glucose medium (0–10 mM) with DHA for 48 h. Subsequently, DHA-induced cell apoptosis was analyzed by Annexin V-FITC/PI staining. As shown in Fig. 6B, Decrease of glucose concentration in medium resulted in a significant enhancement of cell apoptosis induced by DHA in A549 and PC-9 cells. In addition, glucose attenuated inhibition of clonogenic survival by DHA in both cell lines (Fig. 6C). These results hinted that glycolysis reduced cytotoxicity in response to DHA in NSCLC cells.

### DHA combined with the glycolysis inhibitor decreases cell viability and induces apoptosis in NSCLC cells

**Fig 7:**
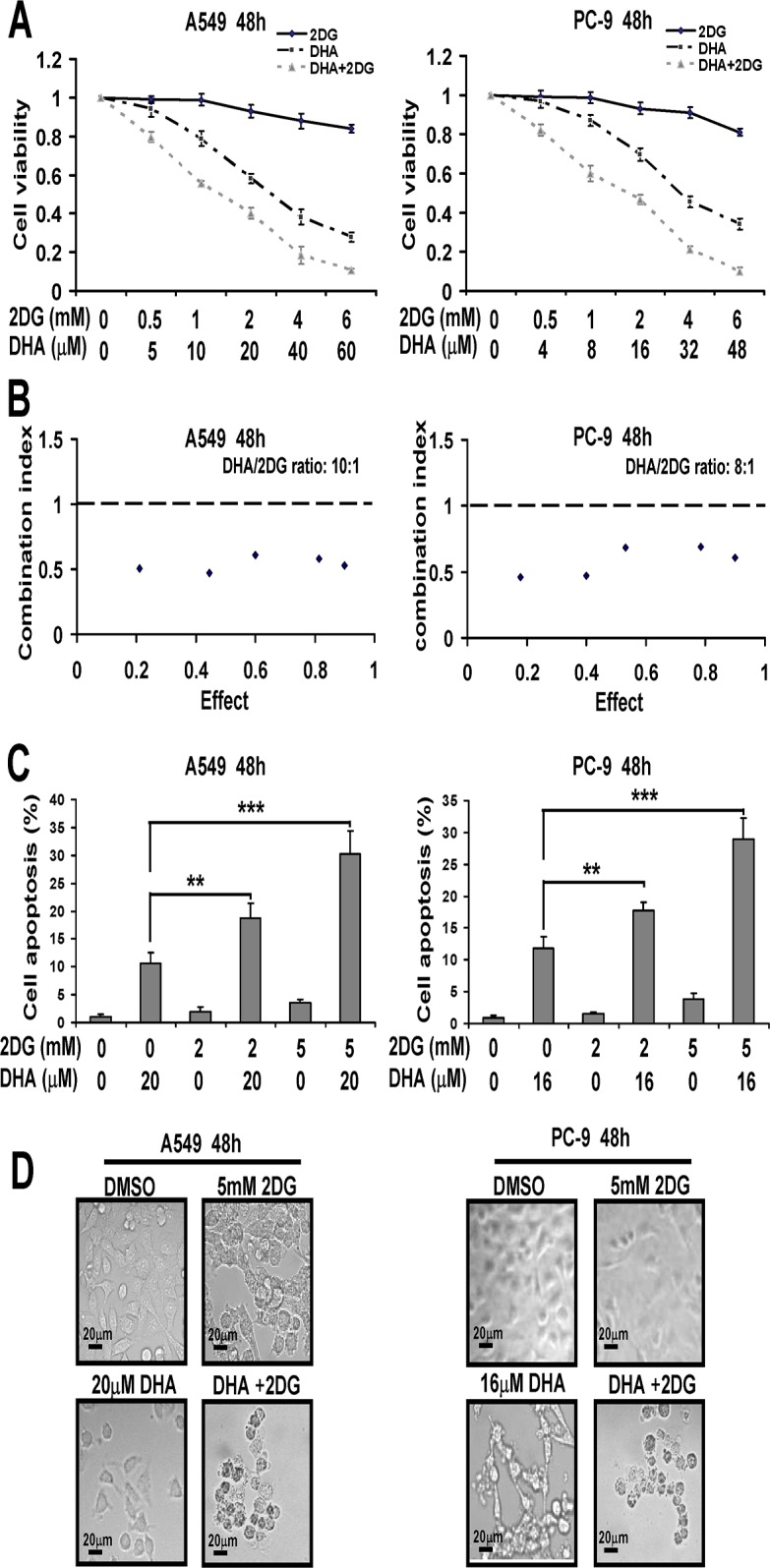
DHA combined with the glycolysis inhibitor decreases cell viability and induces apoptosis in NSCLC cells. (A) Inhibitory effects of DHA combined with 2-Deoxy-D-glucose (2DG) on cell proliferation in A549 and PC-9 cells. Cells were treated with indicated concentrations of DHA and 2DG for 48 h. Cell growth inhibition activity was assessed by MTT. (B) The dose–response curve of each drug was determined and combination index (CI) values for DHA/2DG concentration ratios (10:1 in A549 cells, 8:1 in PC-9 cells) were calculated according to the Chou–Talalay’s method at the 48 h time point, with the biological response being expressed as the fraction of affected cells. Diamond symbol designate the CI value for each fraction affected (effect). CI < 1, CI = 1, CI > 1 indicate synergistic, additive and antagonistic effects, respectively. The effect ranges from 0 (no inhibition) to 1 (complete inhibition). The data are representative of three independent experiments. (C) Influence of DHA combined with 2DG on apoptosis in A549 and PC-9 cells. Cells were treated with indicated concentrations of DHA and 2DG for 48 h. Cell apoptosis was assessed by Annexin V-FITC/PI staining assay. Data were mean ± SD (n = 3). *, *P* < 0.05; **, *P* < 0.01; ***, *P* < 0.001. (D) A549 and PC-9 cells were treated with indicated concentrations of DHA and 2DG for 48 h. Images were recorded using a digital camera with 1280×1280 pixels resolution. Magnification 400. Scale bar: 20 μm.

In this study, we found DHA decreased glycolytic metabolism in NSCLC cells. Moreover, glucose deprivation might sensitize NSCLC cells to DHA. Therefore, we evaluated the effects of glycolysis inhibitor on DHA sensitization. NSCLC cells were treated with DHA plus 2DG, a glucose analog, transiently inhibited glycolysis. We found DHA and 2DG alone reduced cell viability to limited extents, whereas such a loss in cell viability was more pronounced when DHA and 2DG were used in combination (Fig. 7A). These data suggested that DHA and 2DG might synergize to decrease cell viability in NSCLC cells. To confirm this synergism, we treated A549 and PC-9 cells with a combination of the two compounds and calculated the combination index (CI) using Calcusyn software following Chou-Talalay’s method as described under Methods. As shown in Fig. 7B, we showed a synergy between the two agents (CI < 1) in the two NSCLC cells.

In addition, by Annexin V-FITC/PI staining assay, we found that inhibition of glycolysis by use of 2DG resulted in increased cell apoptosis by DHA in both cell lines (Fig. 7C). As shown in Fig. 7D, by microscopic examination, we found 2DG enhanced remarkably the cytotoxicity of DHA. Additionally, microscopic examination of NSCLC cells treated with combinations of DHA and 2DG revealed that treated cells exhibited the classic morphological changes associated with apoptosis including cell shrinkage, blebbing and the formation of apoptotic bodies. These results indicated that glycolysis reduced cytotoxicity in response to DHA in NSCLC cells. Notably, 2DG did not sensitize WI-38 normal lung fibroblast cells to DHA (S1 Fig.).

### DHA plus 2DG induces apoptosis via both extrinsic and intrinsic apoptosis pathways in NSCLC cells

**Fig 8:**
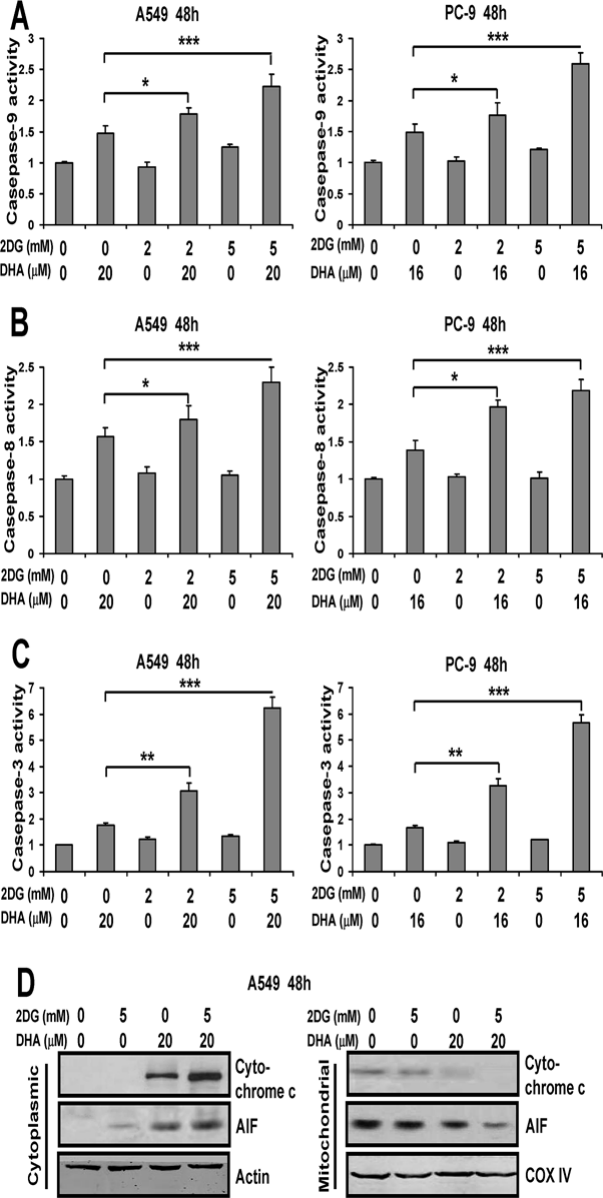
DHA plus 2DG induces apoptosis via both extrinsic and intrinsic apoptosis pathways in NSCLC cells. (A-C) A549 and PC-9 cells were plated, treated for 48 h with the indicated concentrations of DHA and 2DG either alone or in combination. The caspase-9, caspase-8 and caspase-3 activity were quantified as described under Methods. This experiment was repeated thrice. Columns, mean; bars, SD. *, *P* < 0.05; **, *P* < 0.01; ***, *P* < 0.001. (D) A549 and PC-9 cells were treated for 48 h with the indicated concentrations of DHA and 2DG either alone or in combination, fractionated into cytosol and mitochondria, and analyzed for the distribution of cytochrome c and AIF by Western blot analysis. The fractionation quality was verified by the distribution of specific subcellular markers: COX IV for mitochondria and actin for cytosol.

To test whether caspase-8 and/or -9 are involved in DHA combined with 2DG induced apoptosis, fluorogenic substrate cleavage assay was used to detect caspases activity. As shown in Fig. 8A and B, A549 and PC-9 cancer cells treated with two compounds in combination showed significant increase in caspase-9 and caspase-8 activities. Likewise, Fig. 8C showed that DHA plus 2DG increased caspase-3 activity in NSCLC cells, indicating that caspase-8 and -9 acted as the upstream regulators of casapse-3 activation. Moreover, pretreatment with z-IETD-fmk or z-LEHD-fmk, the inhibitors of caspase-8 or -9, dramatically prevented the NSCLC cells apoptosis induced by DHA plus 2DG (data not shown). These results suggested the important roles of caspase-8 and -9 in NSCLC cell apoptosis induced by DHA and 2DG in combination.

Additionally, by Western blotting analysis, we found that 2DG increased cytoplasmic cytochrome c and AIF levels induced by DHA in NSCLC cells (Fig. 8D). In contrast, 2DG combined with DHA decreased mitochondrial cytochrome c and AIF expression in A549 cells (Fig. 8D). Additionally, by Western blotting analysis, significant increase in the amounts of both cytochrome c and AIF of cytoplasm were noted in A549 cells treated with 2DG combined with DHA compared to treatment with single agents (Fig. 8D). In contrast, mitochondrial cytochrome c and AIF levels were reduced in A549 cells treated with two drugs in combination in comparison with single agent. The data shown above indicated DHA plus 2DG induced apoptosis was involved in both extrinsic and intrinsic apoptosis pathways in NSCLC cells.

Recently, numerous studies had shown that the generation of ROS is critical for the toxic effects of DHA. We further investigated whether 2DG enhanced the toxic effects of DHA was involved in upregulation of ROS levels. As shown in S2 Fig., although DHA treatment remarkably increased ROS production, the fact that 2DG treatment did not enhance DHA-induced increase of ROS levels. These results suggested that the ROS production was not involved in the synergistic action of the combination treatment.

## Discussion

In this study, we had investigated the potential of DHA as a novel antitumor drug for NSCLC. Our results indicated that DHA inhibited cell viability, colony formation and induced apoptosis in dose- and time-dependent manner in A549 and PC-9 cells. In addition, we found for the first time that DHA decreased the level of glycolytic metabolism in NSCLC cells. Furthermore, we elucidated DHA-suppressed glycolytic metabolism was associated with inhibition of mTOR activation and GLUT1 expression. Notably, NSCLC cells treated with glycolysis inhibitor 2DG displayed significantly more sensitive to DHA. However, no significant sensitization was observed in WI-38 normal lung fibroblast cells treated with 2DG plus DHA. Moreover, 2DG enhanced DHA-induced cell apoptosis via both extrinsic and intrinsic apoptosis pathways.

Recent studies indicate a potential use of DHA as anticancer agents in many cancer cells both *in vitro* and *in vivo* [3,4,22]. Consistent with previous findings, our data also revealed that DHA exhibited antitumor activities in NSCLC cells *in vitro*. Moreover, we had for the first time shown DHA inhibited NSCLC cells glucose metabolism. mTOR has emerged as a key regulator of cellular metabolism, in addition to its better-known functions in promoting protein synthesis and cell growth [23]. In particular, recent studies suggested that mTOR activation is sufficient to stimulate glucose uptake, glycolysis [24,25]. Previous studies show mTOR activity was suppressed by DHA in ovarian cancer cells [26], and in rhabdomyosarcoma cells [27]. In the present study, we also found DHA inhibited the mTOR activity and glycolytic metabolism in NSCLC cells. Moreover, glucose uptakes were obviously inhibited in A549 cells treated with 40 μM DHA for short time (0–16 h). Notably, no significant apoptosis was induced by DHA for 16 h. These studies suggested DHA refrained glycolytic metabolism was involved in inhibition of mTOR activation not due to the effects of induction of apoptosis. Furthermore, DHA decreased the expression of GLUT1, an important glucose transporter. It has been reported earlier that glucose uptake and GLUT1 levels were prevented by inhibition of mTOR with rapamycin [28]. Additionally, a previous report has indicated GSK-3/TSC2/mTOR pathway regulates GLUT1 expression in vascular smooth muscle cells [29]. Accordingly, this raised a possibility that DHA decreased GLUT1 expression through mTOR pathway, sequentially inhibited glucose uptake.

Cancer cells are frequently subjected to metabolic stress, arising from increased biosynthetic needs and shortage of nutrient supply [30]. Thus, we treated NSCLC cells with low glucose culture or glucose deprivation, which mimicked the simulation of persistent metabolic stress in established NSCLC *in vivo*. Under such circumstances, cells were treated with DHA. As shown in Fig. 6, glucose deprivation alone induced low cytotoxicity in NSCLC cells; however, the combination of DHA and glucose deprivation significantly increased cell death relative to that of DHA alone. Meanwhile, several recent studies reported low uptake and intracellular concentration of glucose may induce ATP depletion and stimulation of mitochondrial death pathway cascade, and may induce oxidative stress and trigger of bax-associated events including the MAPK signal pathway [31,32,33]. Those all suggested that inhibition of glucose uptake by DHA might play a key role in DHA-induced cytotoxicity in NSCLC cells.

Moreover, a key novel finding in our studies is that combination of DHA with the glycolysis inhibitor 2DG, synergistically induced cell death in all two NSCLC cell lines. Whereas, the combination of the two compounds displayed minimal toxicity to WI-38 cells, a normal human colon mucosal epithelial cell line. These results suggested that the synergistic induction of cell death induced by combinations of DHA and 2DG was selective for the NSCLC cells relative to lung epithelial cells. A possible explanation was that the metabolic properties of NSCLC cells are different from those of normal lung epithelial cells [34], and cancer cells are abnormally dependent on aerobic glycolysis. Accordingly, a human cervical cancer cell line HeLa was selected and treated with DHA plus 2DG, Notably, we also found DHA combined with 2DG exerted synergistic effects in cervical cancer HeLa cells (data not shown). This distinction between cancerous and normal cell provided a preliminary indication that a similar combination treatment will not have toxicity issues *in vivo*.

Studies show DHA induces apoptosis by a Bak-dependent intrinsic pathway in Jurkat T-lymphoma cells [35]. DHA induces apoptosis in colorectal cancer cells through the mitochondria-dependent pathway [36]. In contrast, a recent report shows that DHA plus ionizing radiation induces A549 cells apoptosis is involved in the extrinsic but not intrinsic apoptosis pathway [37]. Here, further studies of the mechanism of cytotoxicity induced by DHA plus 2DG obviously indicated that the production of apoptosis was a vital component of this cytotoxicity. In addition, analysis of annexin V and caspase 3/8/9 activities showed that these apoptosis markers were remarkably increased by 2DG in combination with DHA. These results demonstrated that the combination of DHA with 2DG preferentially induced cell death might be due, at least in part, to the induction of apoptosis in NSCLC cells. These findings was consistent with previous studies that certify DHA alone stimulates apoptosis in other tumor cell types [27,38] and that combination of DHA with doxorubicin induces breast cancer cell death via apoptosis [39].

Our investigations provided a more profound insight into apoptosis regulation by DHA plus 2DG. Consistent with previous findings [40], we found that the proapoptotic action of DHA required the activation of caspase-9, caspase-8 and caspase-3. In addition, we showed that both Caspase-8 and Caspase-9 were responsible for the apoptosis triggered by DHA in combination with 2DG. Moreover, the enhanced cytoplasmic cytochrome c and AIF expression induced by the combination treatments were found in NSCLC cells. These indicated that cells apoptosis induced by combination treatments using DHA and glycolysis inhibitors might be involved with extrinsic and mitochondrial apoptotic pathway.

In previous studies, DHA increases cervical cancer HeLa cells and NSCLC A549 cells ROS levels [40,41], which contributes to cell death. Similar studies, consistent with these findings, show that DHA enhances Apo2L/TRAIL-mediated apoptosis in pancreatic cancer cells via ROS-mediated upregulation of death receptor 5 [4,22]. In this study, we also found DHA elicited ROS generation. Notably, treatment with 2DG was not able to significantly increase the amount of ROS caused by the treatment of DHA. These results suggested that DHA induced NSCLC cells apoptosis was involved in ROS; however, ROS was not implicated in the ability of 2DG to enhance DHA-induced apoptosis.

Several studies have shown that a significant number of malignant tumors expressed GLUT1 which is not detected in normal epithelium [42,43]. Increased GLUT1 levels with the high uptakes of glucose are essential for cancer cell to cope with the incremental need of energy [44]. Moreover, high GLUT1 levels have been shown to relieve cell apoptosis through preventing mitochondrial cytochrome c release and downstream caspase activation in B-cell acute lymphoblastic leukemia cells [45]. Consistent with previous studies, our current data showed that GLUT1 overexpression blocked NSCLC cell death induced by DHA. Accordingly, the decrease in GLUT1 expression following DHA treatment, provided DHA a therapeutic edge in NSCLC.

Here we demonstrated for the first time, that regulation of glycolysis by DHA was involved with mTOR pathway in NSCLC cells. Our current findings showed that the use of a glycolysis inhibitor 2DG enhanced the anticancer effect of DHA in the treatment of NSCLC cells *in vitro*. However, further data also needs to be obtained to evaluate the anticancer effect of DHA combined with 2DG *in vivo*.

## Supporting Information

S1 Fig 2DG fails to increase the ROS levels induced by DHA in NSCLC cells. A549 Cells were incubated with indicated concentrations of DHA and 2DG for 48 h. ROS formation was determined on loading of the cells with the oxidation-sensitive dye DCFDA. Columns, mean of three determinations; bars, SD. ns: not significant.
(TIF)

S2 Fig 2DG is incapable of enhancing DHA-induced cytotoxicity in NSCLC cells. WI-38 cells were incubated with indicated concentrations of DHA and 2DG for 48 h. Cell viability was assessed by MTT. Columns, mean of three determinations; bars, SD. ns: not significant.
(TIF)
