# Supplementary figures and images for: Dihydroartemisinin Inhibits Glucose Uptake and Cooperates with Glycolysis Inhibitor to Induce Apoptosis in Non-Small Cell Lung Carcinoma Cells
Source: PLoS One. 2015 Mar 23;10(3):e0120426. doi: 10.1371/journal.pone.0120426 (PMC4370589; doi:10.1371/journal.pone.0120426)

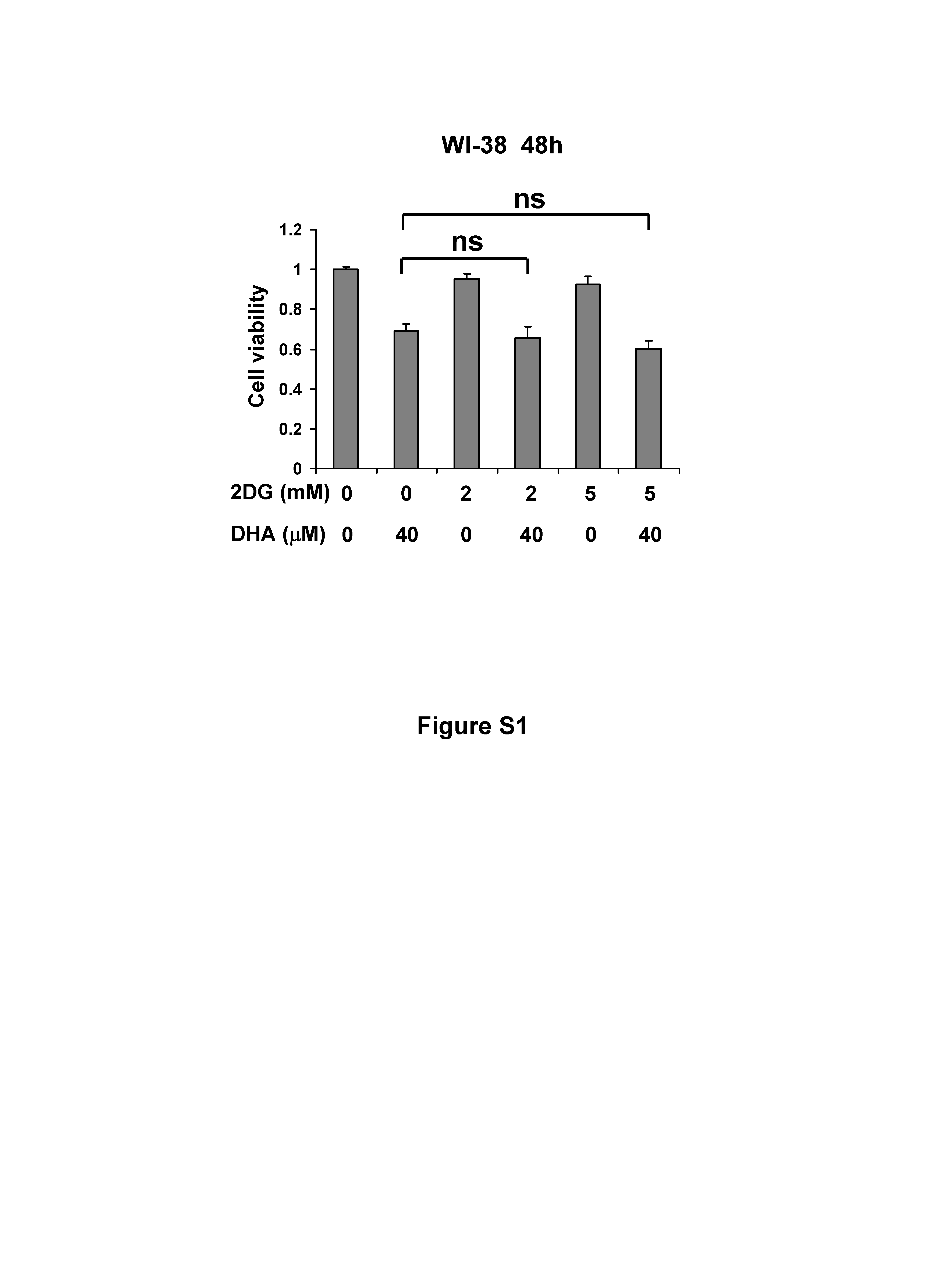

Supplement: S1 Fig — A549 Cells were incubated with indicated concentrations of DHA and 2DG for 48 h. ROS formation was determined on loading of the cells with the oxidation-sensitive dye DCFDA. Columns, mean of three determinations; bars, SD. ns: not significant. (TIF) [file pone.0120426.s001.tif]

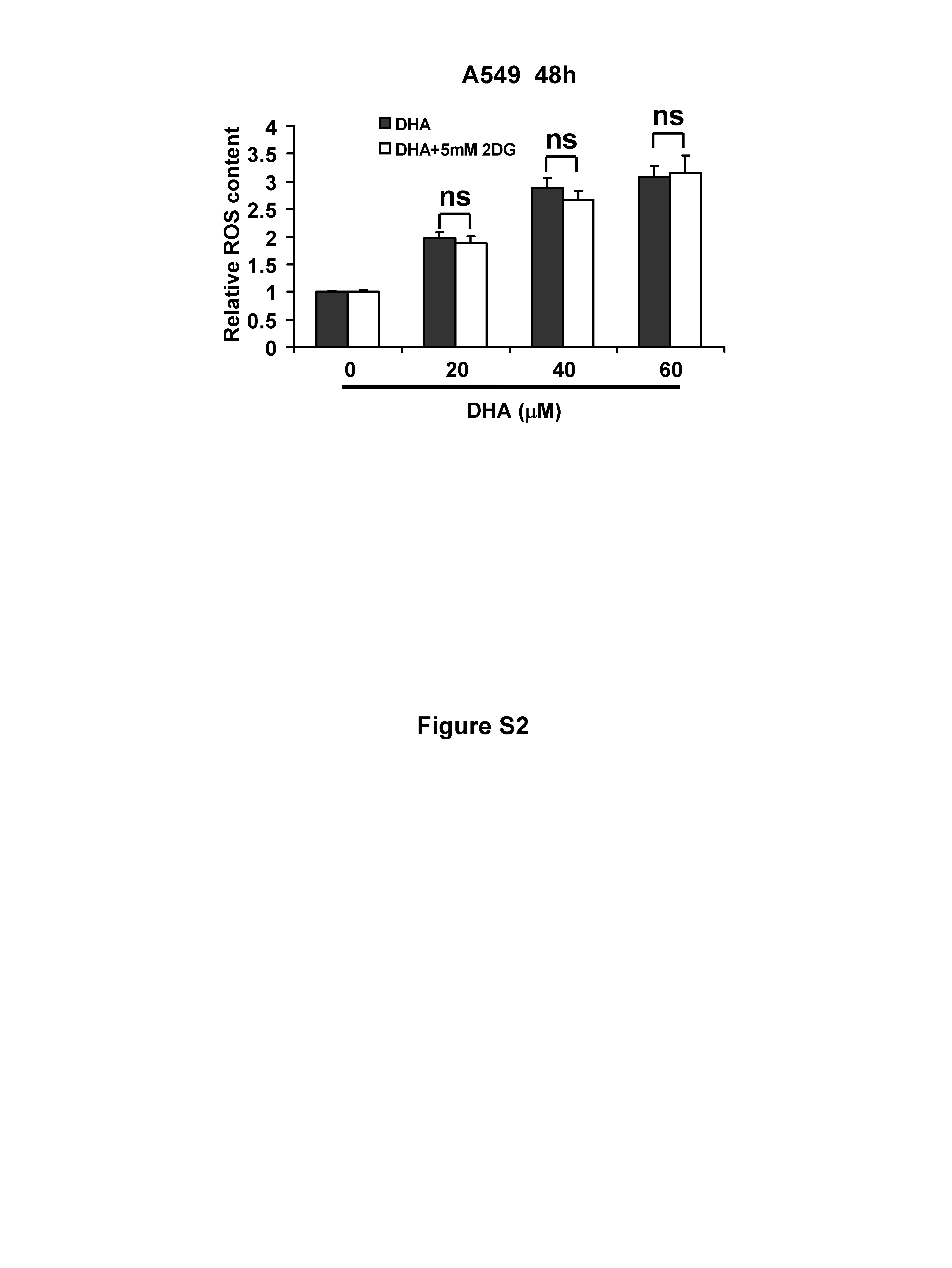

Supplement: S2 Fig — WI-38 cells were incubated with indicated concentrations of DHA and 2DG for 48 h. Cell viability was assessed by MTT. Columns, mean of three determinations; bars, SD. ns: not significant. (TIF) [file pone.0120426.s002.tif]
